# Regulation of Pleiotrophin, Midkine, Receptor Protein Tyrosine Phosphatase β/ζ, and Their Intracellular Signaling Cascades in the Nucleus Accumbens During Opiate Administration

**DOI:** 10.1093/ijnp/pyv077

**Published:** 2015-07-11

**Authors:** Daniel García-Pérez, María Luisa Laorden, María Victoria Milanés

**Affiliations:** Group of Cellular and Molecular Pharmacology, University of Murcia, Campus de Espinardo, Murcia, Spain (Mr García-Pérez, Drs Laorden, and Milanés); IMIB, Instituto Murciano de Investigación Biosanitaria, Murcia, Spain (Mr García-Pérez, Drs Laorden, and Milanés).

**Keywords:** Astrocyte, glial fibrillary acidic protein, midkine, morphine, pleiotrophin, withdrawal

## Abstract

**Background:**

Most classes of addictive substances alter the function and structural plasticity of the brain reward circuitry. Midkine (MK) and pleiotrophin (PTN) are growth/differentiation cytokines which, similarly to neurotrophins, play an important role in repair, neurite outgrowth, and cell differentiation. PTN or MK signaling through receptor protein tyrosine phosphatase β/ζ (RPTPβ/ζ), leads to the activation of extracellular signal-regulated kinases and thymoma viral proto-oncogene. This activation induces morphological changes and modulates addictive behaviors. Besides, there is increasing evidence that during the development of drug addiction, astrocytes contribute to the synaptic plasticity by synthesizing and releasing substances such as cytokines.

**Methods:**

In the present work we studied the effect of acute morphine administration, chronic morphine administration, and morphine withdrawal on PTN, MK, and RPTPβ/ζ expression and on their signaling pathways in the nucleus accumbens.

**Results:**

Present results indicated that PTN, MK, and RPTPβ/ζ levels increased after acute morphine injection, returned to basal levels during chronic opioid treatment, and were up-regulated again during morphine withdrawal. We also observed an activation of astrocytes after acute morphine injection and during opiate dependence and withdrawal. In addition, immunofluorescence analysis revealed that PTN, but not MK, was overexpressed in astrocytes and that dopaminoceptive neurons expressed RPTPβ/ζ.

**Conclusions:**

All these observations suggest that the neurotrophic and behavioral adaptations that occur during opiate addiction could be, at least partly, mediated by cytokines.

## Introduction

The action of many addictive substances converges on the mesolimbic dopaminergic reward pathway, inducing increased firing of dopaminergic neurons in the ventral tegmental area (VTA) of the midbrain and a subsequent increase of dopamine (DA) release in the nucleus accumbens shell (NAc shell; Di Chiara and Imperato, 1988; Ikemoto, 2007). Additionally, drugs of abuse produce widespread effects on the structure and function of neurons throughout the brain reward circuitry, which are believed to underlie the long-lasting behavioral phenotypes that characterize addiction (Russo et al., 2009). However, the molecular mechanisms regulating the neuronal remodeling are not fully understood yet.

The role of glial cells in providing structural, metabolic, and trophic support to neurons has been well established (Kettenmann and Ransom, 1995). Moreover, glial cells are considered the immune-competent cells of the central nervous system (CNS), as well as crucial components of synaptic plasticity (Huang et al., 2000; Ransohoff and Brown, 2012). There is increasing evidence that drugs of abuse produce alterations in CNS immunology. For example, opioids induce profound changes in glial cellular morphology and phenotypic immunohistological marker expression (GFAP, glial fibrillary acidic protein, a cell surface marker of astrocyte reactivity) in specific brain areas (Beitner-Johnson et al., 1993). Importantly, the actions of opioids through glial reactivity are involved in the development of opioid dependence (Hutchinson et al., 2008, 2009; Watkins et al., 2009).

It has been suggested that midkine (MK), a secreted heparin binding growth factor/cytokine (Kadomatsu et al., 1988), and pleiotrophin (PTN), also known as heparin binding-growth associated molecule (HB-GAM; Deuel et al., 2002), could be involved in addiction to drugs of abuse. PTN mRNA and/or MK mRNA levels are up-regulated after acute amphetamine administration (Le Grevès, 2005) and after injection of delta-9-tetrahydrocannabinol (Mailleux et al., 1994) or morphine (Ezquerra et al., 2007) in brain areas related to addiction, such as the NAc shell, the prefrontal cortex, and the hippocampus, respectively. Likewise, increased mRNA and protein levels were found in the prefrontal cortices of alcoholics and tobacco smokers (Flatscher-Bader and Wilce, 2008). Given that these cytokines exert effects that are similar to those of neurotrophins, these findings support the hypothesis that these two cytokines are up-regulated in order to induce neurotrophic or neuroprotective effects during drug consumption (Herradón and Pérez-García, 2013). MK and PTN bind common receptors, including receptor protein tyrosine phosphatase β/ζ (RPTPβ/ζ; Muramatsu, 2002), which is abundantly expressed in the CNS. The interaction of MK or PTN with RPTPβ/ζ establishes a “ligand-dependent inactivation” of RPTPβ/ζ, presumably as a consequence of RPTPβ/ζ dimerization (Deuel et al., 2002). Thus, the signaling of PTN or MK through RPTPβ/ζ leads to the activation of extracellular-signal regulated kinase (ERK) and phosphatidylinositol 3-kinase–thymoma viral proto-oncogene (Akt) (Polykratis et al., 2005; Qi et al., 2001), important axes inducing morphological changes and modulating addictive behaviors.

The present study aimed to identify whether the expression of PTN, MK, RPTPβ/ζ, and their intracellular signaling pathways (Akt and ERK) are altered as a result of acute and chronic morphine exposure and/or morphine withdrawal in the NAc. We further assessed the possible activation of astrocytes, which could lead to the release of astrocyte-related soluble factors. Finally, we also characterized those cell subpopulations that produced and secreted PTN and/or MK and those that expressed RPTPβ/ζ in response to morphine administration or morphine withdrawal.

## Methods

### Subjects

Male Wistar rats (n = 65; Harlan) were adapted to a standard 12h light-dark cycle (lights on: 0800–2000h) for 7 days before the beginning of the experiments. All surgical and experimental procedures were performed in accordance with the European Communities Council Directive of November 24, 1986 (86/609/EEC), and were approved by the local Committees for animal research (REGA ES300305440012).

### Drug Treatment and Experimental Procedure

Rats were implanted subcutaneously (s.c.) with placebo pellets (lactose) for 6 days. Another set of rats were made dependent on morphine by implantation (s.c.) of two 75mg morphine pellets under light ether anesthesia. On day 7, rats were injected intraperitoneally (i.p.) with either morphine HCl (20mg/kg; in a volume of 1ml/kg body weight), naloxone (1mg/kg; 1ml/kg body weight), or an equivalent volume of 0.9% saline, and sacrificed 2h later.

### Electrophoresis and Western Blotting

Samples containing equal quantities of total proteins (20–40mg, depending on the protein of interest) were separated by 6%, 10%, or 12% sodium dodecyl sulfate polyacrylamide gel electrophoresis (depending on the molecular weight of the protein of interest) and transferred onto polyvinylidene difluoride membranes (Millipore). Membranes were blocked in Tris-buffered saline (TBS) containing 0.15 % Tween-20 (TBS-T) and 1% Bovine serum albumin (BSA) for 90 minutes at room temperature (RT), and incubated overnight at 4ºC with the primary antibody diluted in 1% BSA in TBS-T. The following primary antibodies were used: goat polyclonal anti-PTN (1:1000; AF-252-PB, R&D Systems); rabbit polyclonal anti-MK (1:500; sc-20715, Santa Cruz Biotechnology); mouse monoclonal anti-RPTPβ/ζ (1:750; 610180, BD Transduction Laboratories); mouse monoclonal anti-phospho-ERK 1/2 (p-ERK 1/2; 1:1000; sc-7383; Santa Cruz Biotechnology); mouse monoclonal anti-ERK 1/2 (1:1000; sc-135900; Santa Cruz Biotechnology); rabbit monoclonal anti-phospho-Akt (p-Akt; 1:2000; #4060, Cell Signaling Technology Inc.); and rabbit polyclonal anti-Akt (1:1000; #9272, Cell Signaling Technology Inc.). Blots were subsequently reblocked and probed with rabbit polyclonal anti-glyceraldehyde 3-phosphate dehydrogenase (GAPDH) (1:5000; #2118, Cell Signaling Technology Inc.) or rabbit polyclonal anti–α-Tubulin (1:2500; #2144, Cell Signaling Technology Inc.).

### GFAP Immunohistochemistry

Sections of the NAc shell were used for immunohistochemistry to detect astrocytes. Immunohistochemistry was performed as described by García-Pérez et al. (2012). We used mouse monoclonal anti-GFAP (1:400; sc-33673, Santa Cruz Biotechnology) as the primary antibody. The secondary antibody was horse anti-mouse (1:500; BA-2000, Vector Laboratories).

### Quantification GFAP-Positive Cells

Neuroanatomical sites were identified using the Paxinos and Watson (1997) atlas. Photomicrographs were captured by means of a Leica microscope (DM 4000B; Leica) connected to a video camera (DFC290, Leica). GFAP-positive cell nuclei were counted using a computer-assisted image analysis system (QWIN, Leica). Positive cells were counted at 20X magnification. A square field (325 µm) was superimposed upon the captured image to use as a reference area. The number of astrocytes was counted bilaterally in four to five sections from each animal, including the rostral NAc shell and caudal NA(shell), and averaged to obtain a single value for each rat. The whole histological quantification was performed blindly. Total counts for different brain regions are expressed as mean ± standard error of the mean (SEM).

### GFAP Densitometric Analysis

The same conventional light microscopy described above was used for an optical density study of the nuclei and processes as described by García-Pérez et al. (2014). In addition, the area outlined in each image was also calculated to assure that there were no differences between the regions of interest analyzed in different groups.

###  Immunofluorescence

The characterization and specificity of antibodies used in this study have previously been established and proven to be suitable for our research by isotype, epitope, applications, and species reactivity. Negative controls without the primary antibody also were used to assure a lack of non-specific binding of the secondary antibodies used for immunofluorescence (García-Pérez et al., 2013). Sections were treated with citrate buffer (60°C for 20min). Non-specific Fc binding sites were blocked with 2% normal horse serum/0.3% Triton-X-100 in PBS for 1h at RT, and the sections were incubated for 72h (4ºC, constant shaking) with primary antibodies: mouse monoclonal anti-GFAP (1:400; sc-33673, Santa Cruz Biotechnology; Roberts et al., 2009; Wang et al., 2013), goat polyclonal anti-PTN (1:400; AF-252-PB, R&D Systems; Gurok et al., 2004; Marchionini et al., 2007), and rabbit polyclonal anti-MK (1:250; sc-20715, Santa Cruz Biotechnology; Doi et al., 2011; Lorente et al., 2011). Secondary antibodies were applied sequentially for 4 h: Alexa Fluor 488 anti-rabbit immunoglobulin G (IgG) (1:1000; A-21206, Invitrogen), Alexa Fluor 594 anti-goat IgG (1:1000; A-11058, Invitrogen), and Alexa Fluor 405 anti-mouse IgG (1:1000; A-31553, Invitrogen).

Striatal sections containing the NAc shell were stained with mouse monoclonal anti-RPTPβ/ζ (1:50; 610180, BD Transduction Laboratories; Maeda and Noda, 1998; Lorenzetto et al., 2013) and rabbit polyclonal raised against cyclic adenosine monophosphate-regulated phosphoprotein of 32kDa (DARPP-32) phosphorylated at Threonine 34 (p-DARPP-32 Thr-34; 1:400; ab51076, Abcam; Kim et al., 2011; Yuste et al., 2012). Appropriate secondary antibodies were used: Alexa Fluor 488 anti-rabbit IgG (1:1000; A-21206, Invitrogen) and Alexa Fluor 594 anti-mouse IgG (1:1000; A-21203, Invitrogen). Sections were incubated in 4′,6-diamidino-2-phenylindole (DAPI) (1:100 000) for 1min.

### Confocal Analysis

The brain sections were examined using a Leica DMIRE2 confocal microscope and Leica Confocal Software (Leica Microsystems). Images were captured from low magnification to high magnification (20X to 63X oil objective) as previously described by García-Pérez et al. (2015). Confocal images were obtained using 405-nm excitation for Alexa Fluor 405 or DAPI, 488-nm excitation for Alexa Fluor 488, and 543-nm excitation for Alexa Fluor 594.

### Materials

Morphine HCl and morphine base were supplied from Alcaliber Laboratories in cooperation with the Área de Estupefacientes y Psicotropos, Agencia Española del Medicamento y de Productos Sanitarios. Naloxone HCl was purchased from Sigma-Aldrich (Sigma Chemical Co). Morphine HCl and naloxone HCl doses are expressed as the weight of the salt.

### Statistical Analysis

Data are presented as mean ± SEM. Data were analyzed using one-way or two-way analysis of variance (ANOVA) followed by a post hoc Newman–Keuls test. Correlations between changes in protein expression were assessed using the Pearson correlation. Differences with a *p* < 0.05 were considered significant. Statistical analyses were performed with GraphPad Prism 5 (GraphPad Software Inc.).

## Results

### Effects of Acute and Chronic Morphine Administration and Precipitated Morphine Withdrawal on PTN, MK, and RPTPβ/ζ Expression in the NAc Shell

We focused our analysis on the NAc, a brain region that plays an important role in the acute-rewarding morphine effects and in the development of morphine dependence (Di Chiara and Imperato, 1988; Frenois et al., 2002). We studied the NAc shell because this portion of the NAc appears to be more important than the core for reward and receives strong dopaminergic innervation from the posteromedial VTA (Ikemoto, 2007). This experiment addressed whether PTN, MK, or RPTPβ/ζ protein levels were altered after different treatment regimens: (a) pla+mor: rats were implanted with placebo pellets and on day 7 were injected i.p. with an acute dose of morphine; (b) mor+sal: another set of rats were made dependent on morphine by implantation of two morphine pellets, and received saline on day 7; or (c) mor+nx: morphine-dependent rats were injected i.p. with naloxone on day 7, and thus, developed morphine withdrawal.

**Figure 1.**
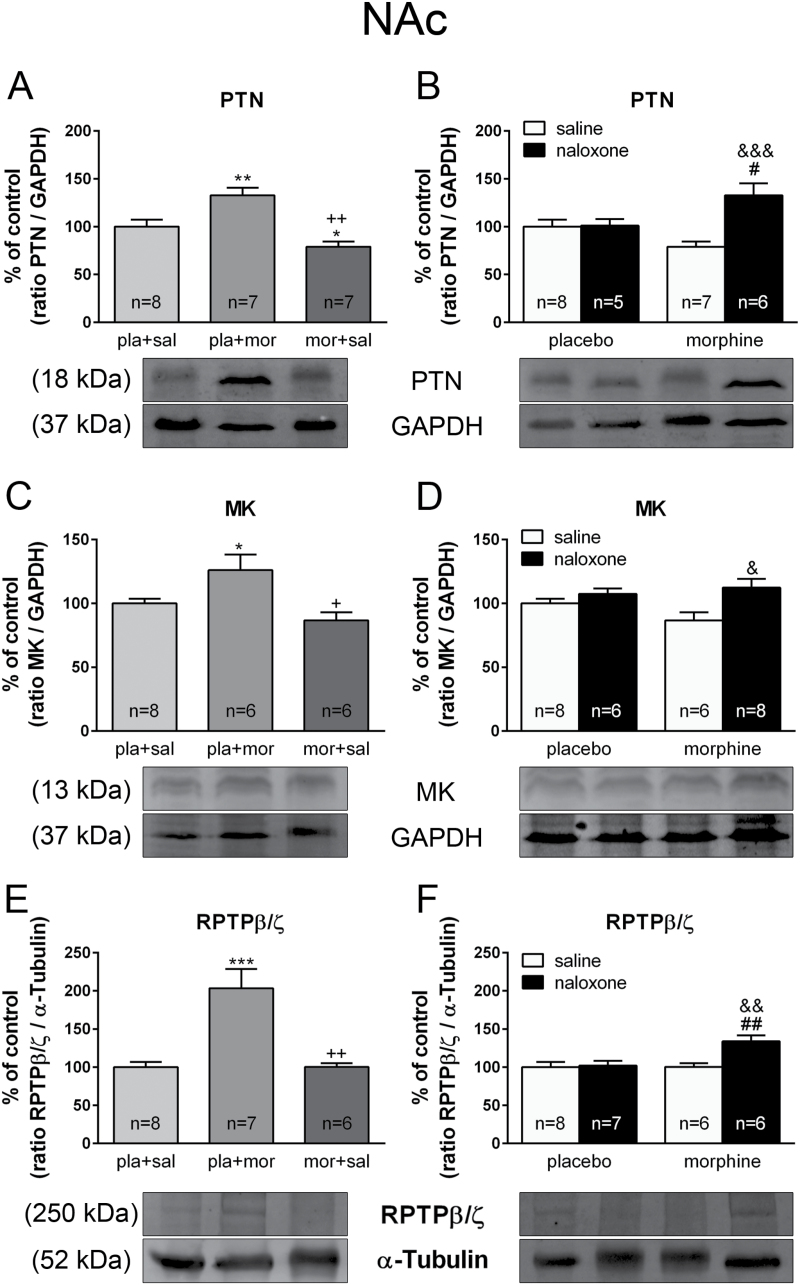
Pleiotrophin (PTN), midkine (MK), and receptor protein tyrosine phosphatase β/ζ (RPTPβ/ζ) protein expression are altered by acute and chronic morphine administration and during morphine withdrawal in the nucleus accumbens (NAc). Over a 7 day period, control (pla) and morphine (mor)-dependent rats received saline (sal), morphine (mor; 20mg/kg i.p.), or naloxone (nx; 1mg/kg s.c.) on day 7 and were sacrificed 2h later. Semi-quantitative analysis and representative immunoblots of PTN (A, B), MK (C, D), and RPTPβ/ζ (E, F) proteins in the NAc were isolated from rats receiving the above treatments. Each bar corresponds to the mean ± standard error of the mean. Values are expressed as a % of controls. ^*^ *p* < 0.05, ^**^ *p* < 0.01, ^***^ *p* < 0.001 vs. pla+sal; ^+^ *p* < 0.01, ^++^ *p* < 0.001 vs. pla+mor; ^#^ *p* < 0.05, ^##^ *p* < 0.01 vs. pla+nx; ^&^ *p* < 0.05, ^&&^ *p* < 0.01, ^&&&^ *p* < 0.001 vs. mor+sal

ANOVA showed significant effects for PTN after morphine administration [*F*(2,21) = 14.250; *p* = 0.0002] and MK [*F*(2,19) = 6.408; *p* = 0.0084] in the NAc shell. As shown in Figure 1A and C, post hoc comparisons showed that acute morphine administration significantly elevated the expression levels of PTN (*p* < 0.01) and MK (*p* < 0.05). Such an increase was not observed during chronic morphine administration (*p* < 0.01) compared with acute morphine injection (PTN: *p* < 0.001; MK: *p* < 0.01). Two-way ANOVA for PTN expression showed a significant effect of acute treatment [*F*(1,22) = 10.50; *p* = 0.0038], and an interaction between pretreatment and acute treatment [*F*(1,22) = 9.68; *p* = 0.0051]. Post hoc tests revealed that PTN levels in the NAc shell were increased after naloxone precipitated morphine withdrawal compared with chronic morphine-treated rats (*p* < 0.001) and with placebo-treated rats receiving saline (*p* < 0.05), as shown in Figure 1B. Two-way ANOVA for MK showed a significant effect of acute naloxone injection [*F*(1,24) = 8.46; *p* = 0.0077]. Post hoc tests revealed that MK levels in the NAc shell were increased after naloxone precipitated morphine withdrawal (*p* < 0.05), as shown in Figure 1D.

Western blot analysis was developed to examine whether morphine and/or morphine withdrawal affected the protein expression levels of RPTPβ/ζ. In the NAc shell, ANOVA showed a significant effect after acute morphine [*F*(2,20) = 14.590; *p* = 0.0002]. As shown in Figure 1E, post hoc comparisons showed that acute morphine administration significantly elevated RPTPβ/ζ (*p* < 0.001) expression. However, there was a decrease in its expression during morphine dependence, compared with acute morphine-treated rats (*p* < 0.01). Two-way ANOVA for RPTPβ/ζ expression revealed main effects for chronic pretreatment [*F*(1,23) = 5.71; *p* = 0.0255], naloxone injection [*F*(1,23) = 6.99; *p* = 0.0145], and a significant interaction between acute and chronic treatment [*F*(1,23) = 5.54; *p* = 0.0276]. Post hoc tests revealed that RPTPβ/ζ levels in the NAc shell were increased in morphine-withdrawn rats compared with morphine-dependent animals receiving saline and with placebo-treated rats receiving naloxone (*p* < 0.01; Figure 1F).

**Figure 2.**
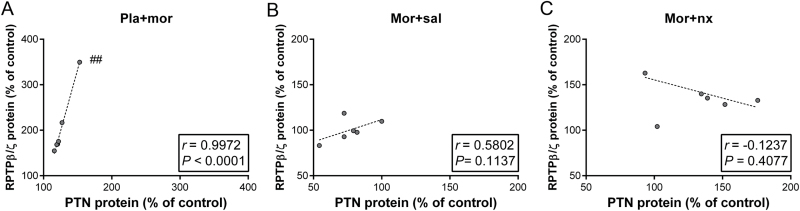
Correlation between pleiotrophin (PTN) and receptor protein tyrosine phosphatase β/ζ (RPTPβ/ζ). The percent increase in PTN levels was positively correlated with RPTPβ/ζ protein after acute morphine injection. No significant correlation was found between PTN expression and RPTPβ/ζ levels during morphine dependence or morphine withdrawal. ^#^ *p* < 0.001: PTN levels vs. RPTPβ/ζ levels.

We next compared the expression of PTN and MK with the induction of RPTPβ/ζ protein levels by Pearson correlation. There were not significant correlations in the different experimental groups between MK expression and RPTPβ/ζ protein levels in the NAc shell (data not shown). In contrast, we observed that after acute morphine administration, the expression of PTN was significantly positively correlated with RPTPβ/ζ levels (Figure 2).

### Astrocytes were Activated by Morphine and Morphine Withdrawal in the NAc Shell

**Figure 3.**
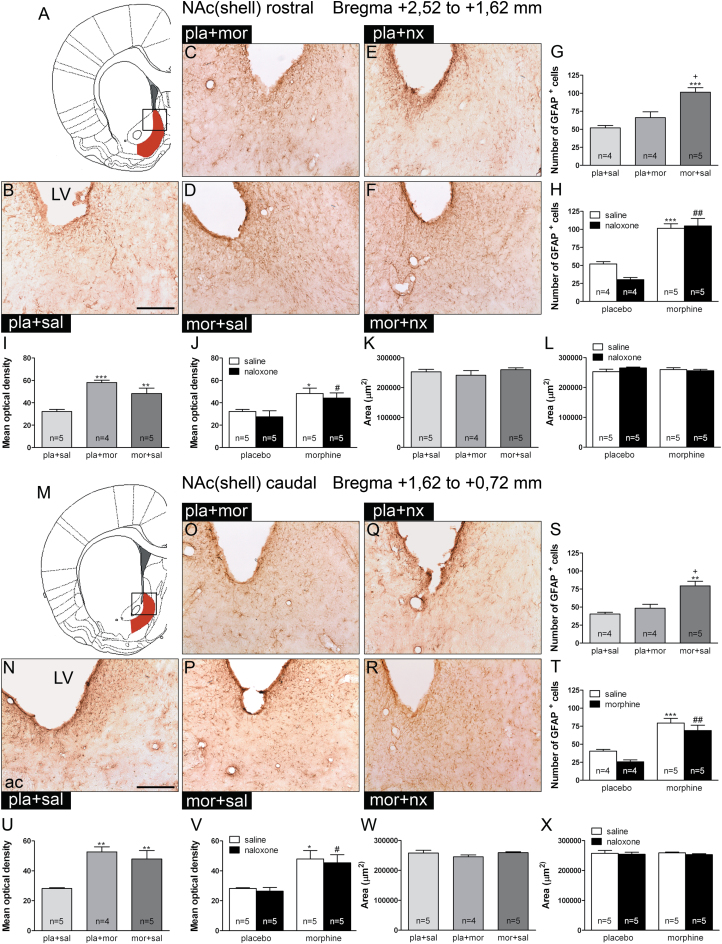
Glial fibrillary acidic protein (GFAP) expression is enhanced by acute and chronic morphine administration and maintained during morphine withdrawal in the nucleus accumbens (NAc) shell, while astrocyte proliferation only occurs in morphine-dependent and morphine-withdrawn rats. Over a 7 day period, control (pla) and morphine (mor)-dependent rats received saline (sal), morphine (mor; 20mg/kg i.p.), or naloxone (nx; 1mg/kg s.c.) on day 7 and were sacrificed 2h later. The analyzed region within the NAc shell rostral and NAc shell caudal is schematically illustrated in A and M, respectively (modificated from Paxinos and Watson, 2007). A rectangle indicates the size of the photomicrographs. Representative photomicrographs showing immunohistochemical detection of GFAP^+^ nuclei and fibers in coronal sections at the NAc shell rostral level (B–F) and NAc shell caudal level (N–R; scale bar: 200 µm). Quantitative analysis of astrocytes in the NAc shell rostral (G, H) and NAc shell caudal (S, T) sections. Mean optical density measurement of GFAP-immunoreactivity in the NAc shell rostral (I, J) and NAc shell caudal (U, V) sections from rats receiving the treatments mentioned above. (K–L, W–X) Reference area used in the densitometric analysis did not differ between groups. LV, lateral ventricle. Each bar corresponds to the mean ± standard error of the mean. ^*^ *p* < 0.05, ^**^ *p* < 0.01, ^***^ *p* < 0.001 vs. pla+sal; ^+^ *p* < 0.01 vs. pla+mor; ^#^ *p* < 0.05, ^##^ *p* < 0.001 vs. pla+nx.

We investigated the possible activation of astrocytes by acute morphine injection, morphine dependence, and/or morphine withdrawal in both rostral and caudal NAc shells. Rostral and caudal NAc shells were examined separately based on studies suggesting a possible dichotomy of their activity according to emotional valence (Reynolds and Berridge, 2002). No differences were found for GFAP-positive cells or GFAP-immunoreactivity (GFAP-IR) between the rostral and caudal NAc shells. ANOVA showed significant effects of morphine administration for GFAP-positive cells [*F*(2,12) = 17.07; *p* = 0.0006] and GFAP-IR [*F*(2,13) = 14.23; *p* = 0.0009] in the rostral NAc shell. Post hoc tests revealed an increase of the number of GFAP-positive cells (*p* < 0.00; Figure 3G) after chronic morphine administration and elevation of GFAP-IR after acute morphine injection (*p* < 0.001) and in morphine-dependent rats (*p* < 0.01; Figure 3I). Two-way ANOVA for the number of GFAP-positive cells revealed significant effects of chronic pretreatment [*F*(1,14) = 73.52; *p* < 0.0001]. Post hoc tests indicated an increase (*p* < 0.001) of GFAP-positive cells during morphine dependence and withdrawal (Figure 3H). Two-way ANOVA for GFAP-IR revealed significant effects of chronic pretreatment [*F*(1,16) = 14.08; *p* = 0.0017]. Post hoc tests indicated an increase (*p* < 0.05) of GFAP-IR during morphine dependence and withdrawal (Figure 3J).

At the NAc shell caudal level, ANOVA showed significant effects of morphine administration for GFAP-positive cells [*F*(2,12) = 15.58; *p* = 0.0008] and GFAP-IR [*F*(2,13) = 11.01; *p* = 0.0024]. Post hoc tests revealed an increase of the number of GFAP-positive cells (*p* < 0.01; Figure 3S) after chronic morphine administration and elevation of GFAP-IR after acute morphine injection and in morphine-dependent rats (*p* < 0.01; Figure 3U). Two-way ANOVA for the number of GFAP-positive cells revealed significant effects of chronic pretreatment [*F*(1,14) = 73.52; *p* < 0.0001] and acute treatment [*F*(1,14) = 4.82; *p* = 0.0456]. Post hoc tests indicated an increase (*p* < 0.001) of GFAP-positive cells during morphine dependence and withdrawal (Figure 3T). Two-way ANOVA for GFAP-IR revealed significant effects of chronic pretreatment [*F*(1,16) = 21.96; *p* = 0.0002]. Post hoc tests indicated an increase (*p* < 0.05) of GFAP-IR during morphine dependence and withdrawal (Figure 3V).

### PTN But Not MK was Overexpressed in Astrocytes During Acute Morphine Administration and Morphine Withdrawal in the NAc Shell

**Figure 4.**
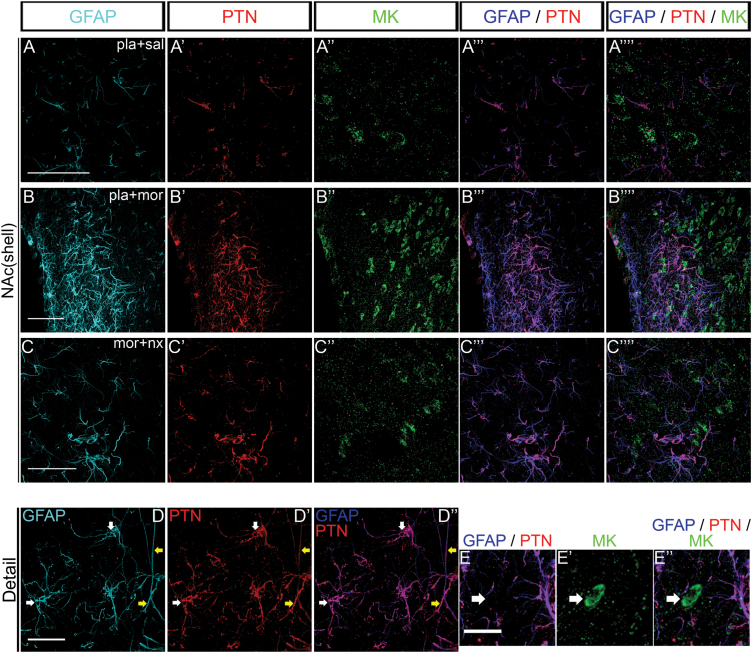
Pleiotrophin (PTN), but not midkine (MK), is overexpressed in astrocytes during acute morphine administration and morphine withdrawal in the nucleus accumbens (NAc) shell. (A–C) Stack of confocal images from the forebrain areas immuno-stained for GFAP (glial fibrillary acidic protein; blue), PTN (red), and MK (green) in control rats rats treated with acute morphine injection or morphine-dependent rats injected with naloxone. (D) High magnification shows that PTN colocalizes with GFAP (activated astrocyte) both in the nuclei (white arrows) and in the processes (yellow arrows) in morphine-withdrawal rats. (E) Panel represents a non-astrocytic cell expressing MK that is surrounded by GFAP^+^ processes containing PTN morphine-withdrawal rats. Scale bar: A–B, 50 µm; C–D, 20 µm

A triple immunofluorescence study revealed that acute morphine (Figure 4B-B’’’’) or morphine withdrawal (Figure 4C-C’’’’) mediated the activation of astrocytes that expressed high levels of PTN protein, but not MK protein in the NAc shell. Colocalization between activated astrocytes and PTN proteins was detected both in the nuclei (white arrows) and in the processes (yellow arrows; Figure 4D-D’’). Figure 4E-E’’ represents a cell that expresses MK and is surrounded by astrocytic processes expressing PTN.

### RPTPβ/ζ was Expressed in Striatal Neurons

**Figure 5.**
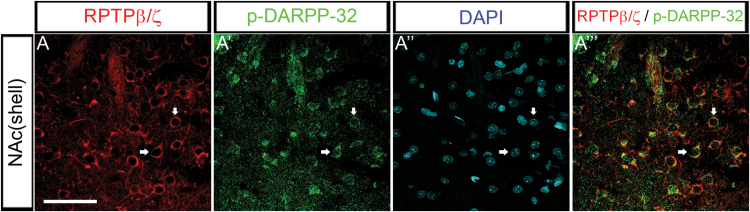
Receptor protein tyrosine phosphatase β/ζ (RPTPβ/ζ) is expressed in striatal neurons. (A) In the nucleus accumbens (NAc) shell of rats injected with acute morphine, RPTPβ/ζ immunoreactivity (red) was distributed homogeneously over the whole structure, on the membranes and proximal projections of neurons. RPTPβ/ζ staining colocalized with some p- cyclic adenosine monophosphate-regulated phosphoprotein of 32kDa Thr-34^+^ neurons (green), confirming its presence on D1R MSNs. 4′,6-Diamidino-2-phenylindole (blue) was used as a counterstaining in both nuclei. Scale bar: A, 50 µm

DARPP-32 is a dual-function protein selectively expressed in all medium-sized spiny neurons (MSNs), and therefore a good marker for MSNs. Moreover, it is well established that acute administration of morphine results in an increase in the state of phosphorylation of DARPP-32 at Thr-34 in the NAc, without affecting phosphorylation at Thr-75, through a DA D1 receptor (D1R)-mediated activation (Borgkvist et al., 2007). Interestingly, we observed in rats injected with acute morphine, that RPTPβ/ζ immunoreactivity in the NAc shell was distributed homogeneously over the whole structure, on the membranes and proximal projections of neurons. RPTPβ/ζ staining colocalized with p-DARPP-32 Thr-34, suggesting its presence on D1R MSNs (Figure 5A-A’’’).

### Effects of Morphine Administration and Precipitated Morphine Withdrawal on Akt and ERK Pathways in the NAc Shell

Previously, PTN or MK signaling has been described as leading to activation of ERK and Akt pathways through RPTPβ/ζ (Qi et al., 2001; Polykratis et al., 2005). In each experiment, the specific signal of p-Akt or p-ERK proteins was normalized to the corresponding Akt or ERK signals, respectively, and then to the level of GAPDH measured in the same preparation.

**Figure 6.**
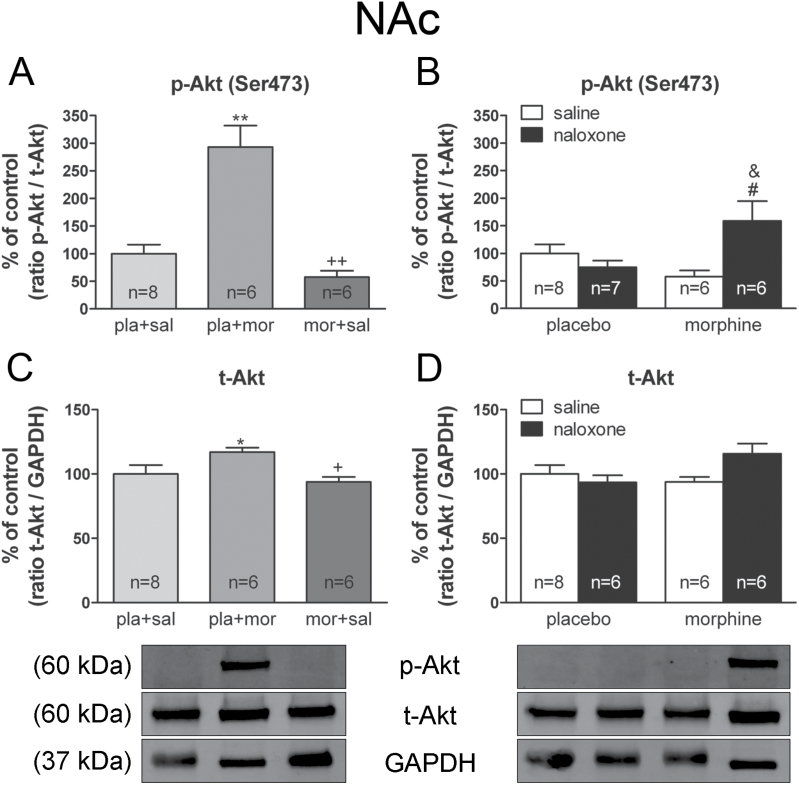
Phospho (p)-thymoma viral proto-oncogene (AKT) levels are enhanced during acute morphine administration and morphine withdrawal in the nucleus accumbens (NAc), while total-Akt (t-AKT) levels only increase during acute opioid injection. Over a 7 day period, control (pla) and morphine (mor)-dependent rats received saline (sal), morphine (mor; 20mg/kg i.p.), or naloxone (nx; 1mg/kg s.c.) on day 7 and were sacrificed 2h later. Semi-quantitative analysis and representative immunoblots of the p-Akt /t-Akt ratio (A, B) and t-Akt levels (C, D) in the NAc were isolated from rats receiving the above treatments. Each bar corresponds to the mean ± standard error of the mean. Values are expressed as a % of controls. ^*^ *p* < 0.05, ^**^ *p* < 0.001 vs. pla+sal; ^+^ *p* < 0.05, ^++^ *p* < 0.001 vs. pla+mor; ^#^ *p* < 0.05 vs. pla+nx; ^&^ *p* < 0.05 vs. mor+sal.

ANOVA for p-Akt [*F*(2,19) = 25.750; *p* < 0.0001] and total-Akt (t-Akt) [*F*(2,19) = 4.457; *p* = 0.0278] showed significant effects after acute morphine administration. Post hoc tests revealed that p-Akt and t-Akt levels were increased after acute morphine injection (*p* < 0.001; *p* < 0.05, respectively; Figure 6A and C), whereas chronic morphine administration decreased both p-Akt (*p* < 0.001) and t-Akt (*p* < 0.05) compared with acute administration of the opiate. Two-way ANOVA for p-Akt revealed an interaction between pretreatment and acute treatment [*F*(1,23) = 9.57; *p* = 0.0051]. Post hoc tests revealed that p-Akt levels in the NAc shell were significantly (*p* < 0.05) elevated in morphine-withdrawn rats compared with the morphine-dependent group receiving saline instead of naloxone and with the placebo-treated rats injected with naloxone (Figure 6B). Two-way ANOVA for t-Akt revealed an interaction between pretreatment and acute treatment [*F*(1,22) = 4.93; *p* = 0.0370], although post hoc tests failed to show any significant effect of chronic morphine or morphine withdrawal (Figure 6D).

**Figure 7.**
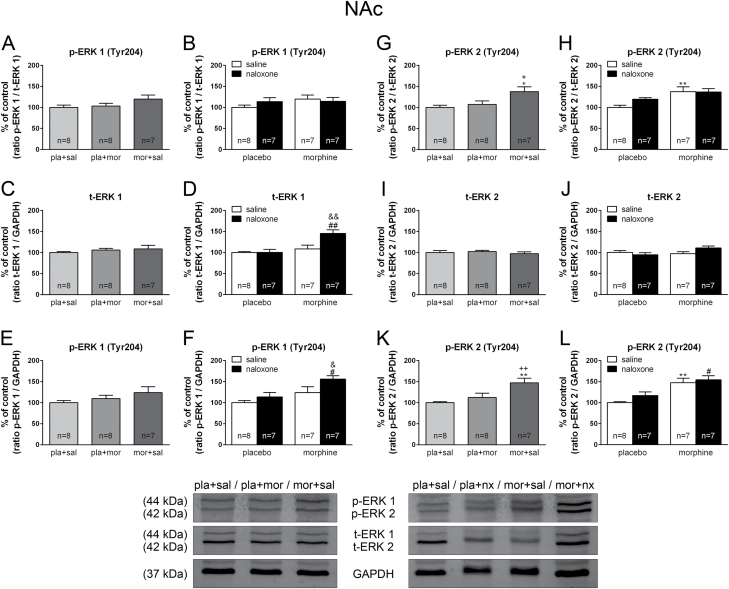
Phospho (p)-extracellular-signal regulated kinase (ERK)/total ERK (t-ERK) ratio, t-ERK levels, and p-ERK absolute levels are altered by acute and chronic morphine administration and during morphine withdrawal in the nucleus accumbens (NAc). Over a 7 day period, control (pla)- and morphine (mor)-dependent rats received saline (sal), morphine (mor; 20mg/kg i.p.), or naloxone (nx; 1mg/kg s.c.) on day 7 and were sacrificed 2h later. Semi-quantitative analysis and representative immunoblots of the p-ERK 1/t-ERK 1 ratio (A, B), t-ERK 1 levels (C, D), p-ERK 1 absolute levels (E, F), p-ERK 2/t-ERK 2 ratio (G, H), t-ERK 2 levels (I, J), and p-ERK 2 absolute levels (K, L) in the NAc were isolated from rats receiving the above treatments. Each bar corresponds to the mean ± standard error of the mean. Values are expressed as % of controls. ^*^ *p* < 0.05, ^**^ *p* < 0.01 vs. pla+sal; ^+^ *p* < 0.05, ^++^ *p* < 0.01 vs. pla+mor; ^#^ *p* < 0.05, ^##^ *p* < 0.001 vs. pla+nx; ^&^ *p* < 0.05, ^&&^ *p* < 0.01 vs. mor+sal.

ANOVA did not show significant effects after acute or chronic morphine for p-ERK 1/total ERK (t-ERK) 1 [*F*(2,22) = 2.043; *p* = 0.1558; Figure 7A], whereas ANOVA for p-ERK 2/t-ERK 2 showed significant effects [*F*(2,22) = 5.284; *p* = 0.0144; Figure 7G]. Post hoc comparisons showed that chronic morphine administration significantly elevated p-ERK 2/ t-ERK 2 (*p* < 0.05) expression in the NAc shell. Two-way ANOVA revealed that morphine pretreatment, acute naloxone injection, or the interaction between pretreatment and acute treatment had no significant effects on p-ERK 1/t-ERK 1 (Figure 7B). Two-way ANOVA for p-ERK 2/t-ERK 2 expression revealed main effects for chronic pretreatment [*F*(1,25) = 13.30; *p* = 0.0012]. Post hoc comparisons showed that chronic morphine administration significantly (*p* < 0.01) elevated p-ERK 2/t-ERK 2 levels compared with the control group (Figure 7H).

Similar results were obtained when considering the p-ERK 1/GAPDH and p-ERK 2/GAPDH ratio. Thus, ANOVA did not show a significant effect after acute morphine for p-ERK 1/GAPDH [*F*(2,22) = 1.553; *p* = 0.2661; Figure 7E]. ANOVA revealed a significant effect for morphine treatment on p-ERK 2/GAPDH [*F*(2,22) = 7.906; *p* = 0.0030]. As shown in Figure 7K, post hoc comparisons showed that acute morphine administration injection significantly elevated p-ERK 2/GAPDH (*p* < 0.01) expression in the NAc shell. Two-way ANOVA showed significant effects of chronic pretreatment [*F*(1,25) = 11.34; *p* = 0.0025] and acute treatment [*F*(1,25) = 5.46; *p* = 0.0278] on p-ERK 1/GAPDH [*F*(1,24) = 12.41; *p* = 0.0017]. As shown in Figure 7F, there was an increase (*p* < 0.01) of p-ERK 1 after naloxone administration to morphine-dependent rats. Two-way ANOVA for p-ERK 2/GAPDH showed significant effects of morphine pretreatment [*F*(1,25) = 25.90; *p* < 0.0001]. Post hoc tests showed that both chronic morphine and morphine withdrawal elevated (*p* < 0.01; *p* < 0.05, respectively) p-ERK 2 levels, compared with their corresponding control groups (Figure 7L).

We next examined the effects of acute morphine administration, chronic morphine administration, and morphine withdrawal on t-ERK 1 (ratio t-ERK 1/GAPDH) and t-ERK 2 (ratio t-ERK 2/GAPDH) protein levels (Figure 7C–J). ANOVA showed no significant effects after acute morphine for t-ERK 1 [*F*(2,22) = 0.6526; *p* = 0.5314] or t-ERK 2 [*F*(2,22) = 0.3751; *p* = 0.6919; Figure 7C and I]. Two-way ANOVA for t-ERK1 expression in the NAc shell showed a significant effect of chronic pretreatment [*F*(1,25) = 14.66; *p* = 0.0008], acute treatment [*F*(1,25) = 7.00; *p* = 0.0139], and an interaction between pretreatment and acute treatment [*F*(1,25) = 6.78; *p* = 0.0153]. As shown in Figure 7D, morphine withdrawal increased t-ERK1 expression compared with morphine-dependent rats (*p* < 0.01) and with placebo-treated rats receiving naloxone (*p* < 0.001). Two-way ANOVA failed to detect any significant effects of chronic morphine pretreatment, acute treatment, or an interaction between pretreatment and acute treatment on t-ERK1 levels in the NAc shell (Figure 7L).

### Relationship Between PTN, MK, and/or RPTPβ/ζ and p-ERK or t-ERK Protein Levels

**Figure 8.**
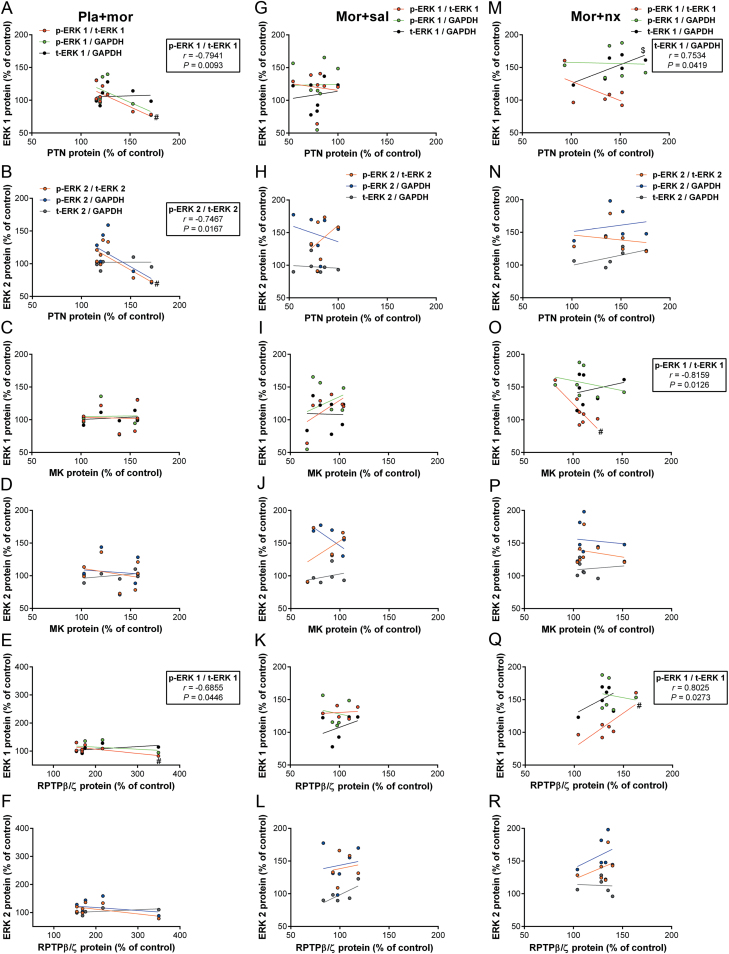
Correlation between pleiotrophin (PTN), midkine (MK), and/or receptor protein tyrosine phosphatase β/ζ (RPTPβ/ζ) and phospho-extracellular-signal regulated kinase (p-ERK) or total ERK (t-ERK) protein levels in different experimental groups. (A–F) After acute morphine injection, significant negative correlations between PTN and RPTPβ/ζ expression and p-ERK 1 and p-ERK 2 protein levels were observed. (M–R) During morphine withdrawal, ERK 1 phosphorylation was positively correlated with RPTPβ/ζ expression, but negatively correlated with MK levels. In addition, a positive relationship between PTN and t-ERK 1 levels in morphine-withdrawn rats is described. ^#^ *p* < 0.05: PTN, MK, or RPTPβ/ζ levels vs. p-ERK 1/t-ERK 1 or p-ERK 2/t-ERK 2 levels. ^$^ *p* < 0.05: PTN levels vs. t-ERK 1/glyceraldehyde 3-phosphate dehydrogenase..

We first compared the expression of PTN, MK, and/or RPTPβ/ζ with the induction of p-ERK or t-ERK protein levels in control rats that were injected with an acute dose of morphine. There were significant negative correlations between PTN expression and p-ERK 1 and p-ERK 2 protein levels in the NAc shell (Figure 8A and B). Moreover, RPTPβ/ζ expression in acute morphine-injected rats was also negatively correlated with changes on p-ERK 1 (Figure 8E). On the other hand, when we investigated the group of rats that were exposed to chronic morphine we did not identify any significant correlation in the expression of these proteins (Supplementary Table S1). During morphine withdrawal, we observed that ERK 1 phosphorylation was positively correlated with RPTPβ/ζ expression (Figure 8Q), but negatively correlated with MK levels (Figure 8O). In addition, we detected a positive relationship between PTN and t-ERK 1 levels in morphine-withdrawn rats (Figure 8M).

However, the correlation analysis performed between PTN, MK, or RPTPβ/ζ and Akt in the NAc during the different morphine paradigms employed, showed that Akt phosphorylation was unrelated with the protein expression levels of these cytoquines or their receptor (Supplementary Table S1).

## Discussion

The present study showed that a single dose of morphine and morphine withdrawal increased the protein levels of PTN and MK in the NAc, but chronic morphine administration had the opposite effect, since protein levels returned to basal levels or even decreased. Our results are supported by previous findings showing that acute morphine administration or withdrawal, but not chronic morphine administration, promote the expression of pro-inflammatory cytokines (Campbell et al., 2013). Moreover, we detected that PTN up-regulation was restricted to astrocytes, as it has been previously described in the literature (Iseki et al., 2002; Mourlevat et al., 2005; Taravini et al., 2011). In contrast, we observed that MK was labeled in non-astrocytic cells. Since astrocytes can also express MK after kainic acid injection (Kim et al., 2010), it could be hypothesized that, depending on the nature of the insult or the damage/cell death it may produce, MK can be over-expressed by neurons or astrocytes.

We also found that the expression of the PTN- and MK-target receptor (RPTPβ/ζ) was regulated in the same way as these cytokines by morphine administration. Although MK and PTN bind in a similar manner to RPTPβ/ζ (Maeda et al., 1996, 1999), the mechanisms triggered by the formation of the complex PTN/ RPTPβ/ζ are better known. The interaction of RPTPβ/ζ with PTN inactivates the intrinsic tyrosine phosphatase activity of RPTPβ/ζ (Meng et al., 2000). We observed that PTN direct RPTPβ/ζ-inactivation after acute morphine injection induced RPTPβ/ζ expression, which could be viewed as a homeostatic response to preserve the regulation of cellular protein tyrosine phosphorylation levels. However, the correlation between PTN and RPTPβ/ζ expression did not occur in morphine-dependent rats. Regarding the RPTPβ/ζ signal, we found a staining pattern in neurons. Our results are in line with previous studies, where this protein was found to be located in neurons but not in astrocytes (Hayashi et al., 2005; Lorenzetto et al., 2013). It is well established that MSNs in the striatum of normal rats express RPTPβ/ζ (Ferrario et al., 2008). Moreover, our findings demonstrate that RPTPβ/ζ is labeled in p-DARPP-32 Thr-34 MSNs. The ability of morphine to stimulate Thr34 phosphorylation is dependent on D1R, according to Borgkvist et al. (2007). Thus, our data suggest that RPTPβ/ζ is expressed in the D1R subtype of MSNs, which are dopaminoceptive neurons that become directly activated after morphine administration and DA release. This expression pattern supports our hypothesis of an interaction between glial and neuronal function during morphine administration and withdrawal.

Astrocytes can display both hypertrophy and proliferation upon treatment with drugs of abuse. Daily morphine administration, using a regimen that produced tolerance and dependence, increased GFAP immunostaining in different brain regions, including the Nac (Garrido et al., 2005). Repeated morphine exposure has also been reported to increase the GFAP mRNA and protein levels in the striatum (Marie-Claire et al., 2004). We also observed an enhancement in GFAP-IR in the rostral and caudal NAc shell during acute morphine administration, chronic morphine administration, and morphine withdrawal. Morphine and opioid signaling have been shown to promote proliferation of astroglia in the postnatal brain (Sargeant et al., 2008). We observed rapid astrocyte proliferation in the VTA (data not shown), the brain area where the rewarding properties of morphine are believed to be firstly mediated. Secondly, this signal causes disinhibition of dopaminergic neuron activity and increases DA release in the NAc shell, where we showed a slower astrocytic proliferation only after chronic morphine administration. In addition, the rostral and caudal NAc shells were examined separately based on a study suggesting a possible dichotomy of their activity according to emotional valence (Reynolds and Berridge, 2002). Although we could not find any significant differences, we observed that the number of astrocytes tended to increase in the rostral NAc shell during morphine withdrawal, a subregion involved in negative emotional valence during withdrawal. On the other hand, positive or rewarding stimuli seem to be associated with activity in the caudal NAc shell, where astrocytes tended to decrease in morphine-withdrawn rats.

Several findings support the idea that central immune signaling contributes substantially to the pharmacodynamic actions of drugs of abuse, since different glial cell modulators are hypothesized to decrease the rewarding effects of opioids. For instance, ibudilast co-administered with morphine significantly reduced the magnitude of opioid-induced dopamine release in the NAc (Bland et al., 2009). Propentofylline also blocked the development of the effects of morphine in the conditioned place preference procedure (Narita et al., 2006). Interestingly, a role for MK and PTN in modulating drug reward behaviors has also been proposed. For example, morphine- and ethanol-induced conditioned place preference (CPP) was augmented in PTN-/- mice compared to PTN+/+ mice (Herradón and Pérez-García, 2013; Vicente-Rodriguez et al., 2014). So, our increased PTN and MK levels in the NAc after an acute morphine dose may be important in limiting the acute rewarding effects of opiates.

Other cytokines have also been implicated in withdrawal-related behavior. Recent findings strongly support the potential role of glial modulators, specifically ibudilast and minocycline, to decrease opioid withdrawal symptoms (Hutchinson et al., 2009, 2010). Moreover, corticotrophin releasing factor and cytokines work together to worsen ethanol withdrawal phenotypes (Knapp et al., 2011). Although the role of PTN and MK in conditioned place aversion remains uncharacterized, future studies should address this issue, since robustly increased levels of PTN and MK were also observed during morphine withdrawal.

When we studied the intracellular targets of PTN/MK signaling, we observed an up-regulation of ERK phosphorylation levels only during chronic morphine treatments (morphine-dependent and morphine-withdrawn rats) in the NAc, which agrees with previous data describing that p-ERK levels in the NAc do not change 60min after acute morphine injection (Muller and Unterwald, 2004). Interestingly, during acute morphine administration, PTN and RPTPβ/ζ levels were negatively correlated with the phosphorylation levels of ERK 1/2. Given the prominent role of p-ERK 1/2 in the NAc in mediating morphine CPP (Lv et al., 2015) or contextual memory (Xu et al., 2012), and the importance of PTN on limiting morphine or ethanol reward (Herradón and Pérez-García, 2013; Vicente-Rodriguez et al., 2014), it is tempting to speculate that PTN limits the rewarding effects of acute morphine through limiting ERK 1/2 phosphorylation. In contrast, during morphine withdrawal, MK and RPTPβ/ζ exerted opposing roles on regulating p-ERK 1. Although RPTPβ/ζ is induced in morphine-withdrawn rats, protein levels do not reach the ones observed in rats subjected to an acute morphine injection. According to this, high PTN protein levels during morphine withdrawal may inactivate the intrinsic tyrosine phosphatase activity of RPTPβ/ζ (Meng et al., 2000), leading to increased p-ERK 1 protein.

We also detected that t-ERK levels could be altered in morphine-dependent rats. In agreement with our results, total ERK1 and ERK2 levels were increased selectively in caudate/putamen after chronic morphine treatment (Ortiz et al., 1995). Interestingly, we detected that PTN may be related to the increased t-ERK1 observed in morphine-withdrawn rats. In contrast, p-Akt levels were increased in the NAc after acute morphine injection and morphine withdrawal, which adds further evidence to the results obtained by Muller and Unterwald (2004). However, the control of Akt phosphorylation in the NAc during morphine administration seems to be independent of PTN, MK, or RPTPβ/ζ, since we did not find any correlation.

In summary, given that PTN, MK, and RPTPβ/ζ levels increase after acute morphine injection, return to basal levels during chronic opioid treatment, and are up-regulated again during morphine withdrawal in the NAc, we hypothesize a role for these cytokines in mediating, at least in part, neurotrophic and behavioral adaptations that are observed during opiate addiction.

## Supplementary Material

For supplementary material accompanying this paper, visit http://www.ijnp.oxfordjournals.org/

## Statement of Interest

The authors declare that they have no conflicts of interest.

## Supplementary Material

Supplementary Table S1
