# Supplementary material for: Regulation of Pleiotrophin, Midkine, Receptor Protein Tyrosine Phosphatase β/ζ, and Their Intracellular Signaling Cascades in the Nucleus Accumbens During Opiate Administration
Source: Int J Neuropsychopharmacol. 2015 Jul 11;19(1):pyv077. doi: 10.1093/ijnp/pyv077 (PMC4772269; doi:10.1093/ijnp/pyv077)
Supplement: Supplementary Table S1 [file ijnp_pyv077_index.html]

Supplementary Data | International Journal of Neuropsychopharmacology

## Supplementary Data

Data files

- Supplementary Data - Supplementary Data
- Supplementary Data - Supplementary Data
- Supplementary Data - Supplementary Data
