# Supplementary material for: Regulation of Pleiotrophin, Midkine, Receptor Protein Tyrosine Phosphatase β/ζ, and Their Intracellular Signaling Cascades in the Nucleus Accumbens During Opiate Administration
Source: Int J Neuropsychopharmacol. 2015 Jul 11;19(1):pyv077. doi: 10.1093/ijnp/pyv077 (PMC4772269; doi:10.1093/ijnp/pyv077)
Supplement: Supplementary Table S1 [file Supplementary_Table_S1.docx]

|  | Pla+mor | Mor+sal | Mor+nx |
| --- | --- | --- | --- |
| PTN vs. | **p-ERK 1 / t-ERK 1**  r = -0.7941  P = 0.0093^#^  **p-ERK 1 / GAPDH**  r = -0.5969  P = 0.0591  **t-ERK 1 / GAPDH**  r = 0.0801  P = 0.4252 | **p-ERK 1 / t-ERK 1**  r = -0.1155  P = 0.4026  **p-ERK 1 / GAPDH**  r = 0.0107  P = 0.4909  **t-ERK 1 / GAPDH**  r = 0.1425  P = 0.3803 | **p-ERK 1 / t-ERK 1**  r = -0.5948  P = 0.1065  **p-ERK 1 / GAPDH**  r = -0.0413  P = 0.4690  **t-ERK 1 / GAPDH**  r = 0.7534  P = 0.0419^$^ |
|  | **p-ERK 2 / t-ERK 2**  r = -0.7467  P = 0.0167^#^  **p-ERK 2 / GAPDH**  r = -0.5990  P = 0.0583  **t-ERK 2 / GAPDH**  r = 0.0102  P = 0.4904 | **p-ERK 2 / t-ERK 2**  r = 0.4013  P = 0.1861  **p-ERK 2 / GAPDH**  r = -0.2543  P = 0.2911  **t-ERK 2 / GAPDH**  r = -0.1026  P = 0.4134 | **p-ERK 2 / t-ERK 2**  r = -0.1775  P = 0.3683  **p-ERK 2 / GAPDH**  r = 0.1998  P = 0.3521  **t-ERK 2 / GAPDH**  r = 0.6232  P = 0.0931 |
| MK vs. | **p-ERK 1 / t-ERK 1**  r = -0.0397  P = 0.4663  **p-ERK 1 / GAPDH**  r = 0.0273  P = 0.4768  **t-ERK 1 / GAPDH**  r = 0.2246  P = 0.3141 | **p-ERK 1 / t-ERK 1**  r = 0.5755  P = 0.1160  **p-ERK 1 / GAPDH**  r = 0.2633  P = 0.3071  **t-ERK 1 / GAPDH**  r = -0.0286  P = 0.4757 | **p-ERK 1 / t-ERK 1**  r = -0.8159  P = 0.0126^#^  **p-ERK 1 / GAPDH**  r = -0.3022  P = 0.2550  **t-ERK 1 / GAPDH**  r = 0.2532  P = 0.2919 |
|  | **p-ERK 2 / t-ERK 2**  r = -0.2627  P = 0.2846  **p-ERK 2 / GAPDH**  r = -0.1094  P = 0.4077  **t-ERK 2 / GAPDH**  r = 0.4699  P = 0.1436 | **p-ERK 2 / t-ERK 2**  r = 0.4894  P = 0.1623  **p-ERK 2 / GAPDH**  r = -0.6224  P = 0.0935  **t-ERK 2 / GAPDH**  r = 0.3157  P = 0.2711 | **p-ERK 2 / t-ERK 2**  r = -0.1988  P = 0.3345  **p-ERK 2 / GAPDH**  r = -0.0964  P = 0.4185  **t-ERK 2 / GAPDH**  r = 0.1713  P = 0.3567 |
| RPTPβ/ζ vs. | **p-ERK 1 / t-ERK 1**  r = -0.6855  P = 0.0446^#^  **p-ERK 1 / GAPDH**  r = -0.2690  P = 0.2799  **t-ERK 1 / GAPDH**  r = 0.5140  P = 0.1190 | **p-ERK 1 / t-ERK 1**  r = 0.1216  P = 0.4228  **p-ERK 1 / GAPDH**  r = -0.1444  P = 0.4084  **t-ERK 1 / GAPDH**  r = 0.3555  P = 0.2785 | **p-ERK 1 / t-ERK 1**  r = 0.8025  P = 0.0273^#^  **p-ERK 1 / GAPDH**  r = -0.1540  P = 0.3854  **t-ERK 1 / GAPDH**  r = 0.5177  P = 0.1464 |
|  | **p-ERK 2 / t-ERK 2**  r = -0.5737  P = 0.0840  **p-ERK 2 / GAPDH**  r = -0.2868  P = 0.2664  **t-ERK 2 / GAPDH**  r = 0.5338  P = 0.1086 | **p-ERK 2 / t-ERK 2**  r = 0.1406  P = 0.4108  **p-ERK 2 / GAPDH**  r = 0.1296  P = 0.4033  **t-ERK 2 / GAPDH**  r = 0.7292  P = 0.0810 | **p-ERK 2 / t-ERK 2**  r = 0.3732  P = 0.2331  **p-ERK 2 / GAPDH**  r = 0.3822  P = 0.2273  **t-ERK 2 / GAPDH**  r = -0.0637  P = 0.4523 |

**Supplementary Table S1:** Correlation analysis between PTN, MK and/or RPTPβ/ζ and p-ERK or t-ERK protein levels in different experimental groups. ^#^*p* < 0.05: PTN, MK or RPTPβ/ζ levels vs. p-ERK 1 / t-ERK 1 or p-ERK 2 / t-ERK 2 levels. ^$^*p* < 0.05: PTN levels vs. t-ERK 1 / GAPDH.
