# Supplementary figures and images for: Regulation of Pleiotrophin, Midkine, Receptor Protein Tyrosine Phosphatase β/ζ, and Their Intracellular Signaling Cascades in the Nucleus Accumbens During Opiate Administration
Source: Int J Neuropsychopharmacol. 2015 Jul 11;19(1):pyv077. doi: 10.1093/ijnp/pyv077 (PMC4772269; doi:10.1093/ijnp/pyv077)

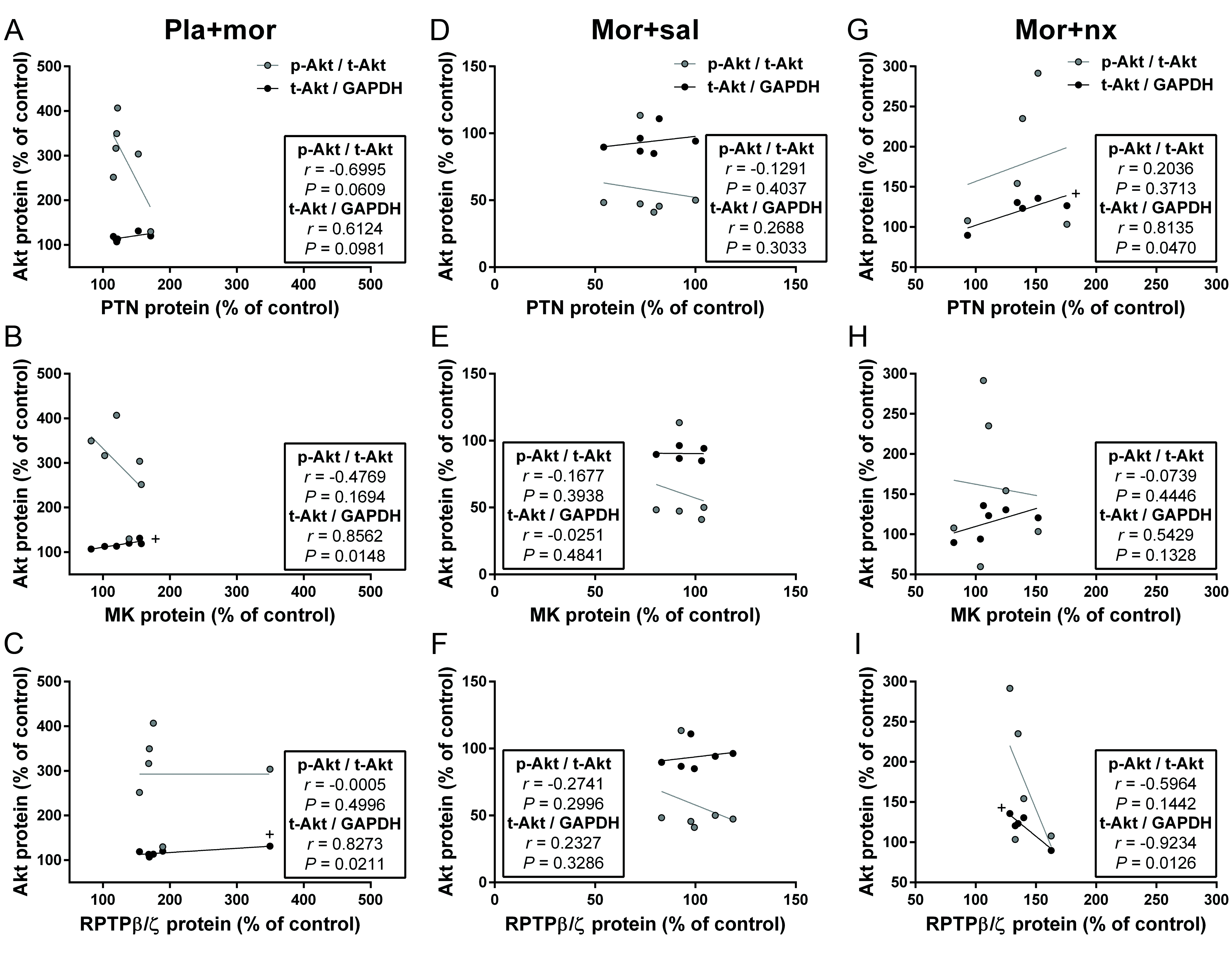

Supplement: Supplementary Table S1 [file Supplementary_Figure_S1.tif]
