# Supplementary material for: Regulation of Pleiotrophin, Midkine, Receptor Protein Tyrosine Phosphatase β/ζ, and Their Intracellular Signaling Cascades in the Nucleus Accumbens During Opiate Administration
Source: Int J Neuropsychopharmacol. 2015 Jul 11;19(1):pyv077. doi: 10.1093/ijnp/pyv077 (PMC4772269; doi:10.1093/ijnp/pyv077)
Supplement: Supplementary Table S1 [file Supplementary_Figure_Legend.docx]

**Supplementary Figure S1** Correlation between PTN, MK and/or RPTPβ/ζ and p-Akt or t-Akt protein levels in different experimental groups. There is no statistical correlation between PTN, MK and/or RPTPβ/ζ expression and p-Akt levels in none of the different morphine administration protocols. However, t-Akt levels seem to be differently correlated with PTN, MK and/or RPTPβ/ζ depending on the experimental group. ^+^*p* < 0.05: PTN, MK or RPTPβ/ζ levels vs. t-Akt / GAPDH.
